# The Therapeutic and Prophylactic Potential of Quercetin against COVID-19: An Outlook on the Clinical Studies, Inventive Compositions, and Patent Literature

**DOI:** 10.3390/antiox11050876

**Published:** 2022-04-29

**Authors:** Mohd Imran, Hamdy Khamees Thabet, Saleh I. Alaqel, Abdullah R. Alzahrani, Abida Abida, Mohammed Kanan Alshammari, Mehnaz Kamal, Anupama Diwan, Syed Mohammed Basheeruddin Asdaq, Sultan Alshehri

**Affiliations:** 1 Department of Pharmaceutical Chemistry, Faculty of Pharmacy, Northern Border University, Rafha 91911, Saudi Arabia; saleh.alaqel@nbu.edu.sa (S.I.A.); abeda.mohammed@nbu.edu.sa (A.A.); 2 Department of Chemistry, Faculty of Arts and Science, Northern Border University, Rafha 91911, Saudi Arabia; hamdykhamees@gmail.com; 3 Department of Pharmacology and Toxicology, Faculty of Medicine, Umm Al-Qura University, Al-Abidiyah, Makkah 21955, Saudi Arabia; aralzahrani@uqu.edu.sa; 4 Department of Pharmaceutical Care, Rafha Central Hospital, North Zone, Rafha 91911, Saudi Arabia; mokaalshammari@moh.gov.sa; 5 Department of Pharmaceutical Chemistry, College of Pharmacy, Prince Sattam Bin Abdulaziz University, Al-Kharj 11942, Saudi Arabia; m.uddin@psau.edu.sa; 6 School of Pharmaceutical Sciences, Apeejay Stya University, Sohna-Palwal Road, Sohna 122103, India; anupama.diwan@asu.apeejay.edu; 7 Department of Pharmacy Practice, College of Pharmacy, AlMaarefa University, Riyadh 13713, Saudi Arabia; 8 Department of Pharmaceutics, College of Pharmacy, King Saud University, Riyadh 11451, Saudi Arabia; salshehri1@ksu.edu.sa

**Keywords:** quercetin, antioxidant, COVID-19, clinical trial, invention, patent

## Abstract

Quercetin is a phenolic flavonol compound with established antioxidant, anti-inflammatory, and immuno-stimulant properties. Recent studies demonstrate the potential of quercetin against COVID-19. This article highlighted the prophylactic/therapeutic potential of quercetin against COVID-19 in view of its clinical studies, inventions, and patents. The literature for the subject matter was collected utilizing different databases, including PubMed, Sci-Finder, Espacenet, Patentscope, and USPTO. Clinical studies expose the potential of quercetin monotherapy, and also its combination therapy with other compounds, including zinc, vitamin C, curcumin, vitamin D3, masitinib, hydroxychloroquine, azithromycin, and ivermectin. The patent literature also examines claims that quercetin containing nutraceuticals, pharmaceuticals, and dietary supplements, alone or in combination with other drugs/compounds, including favipiravir, remdesivir, molnupiravir, navitoclax, dasatinib, disulfiram, rucaparib, tamarixin, iota-carrageenan, and various herbal extracts (aloe, poria, rosemary, and sphagnum) has potential for use against COVID-19. The literature reveals that quercetin exhibits anti-COVID-19 activity because of its inhibitory effect on the expression of the human ACE2 receptors and the enzymes of SARS-CoV-2 (MPro, PLPro, and RdRp). The USFDA designated quercetin as a “Generally Recognized as Safe” substance for use in the food and beverage industries. It is also an inexpensive and readily available compound. These facts increase the possibility and foreseeability of making novel and economical drug combinations containing quercetin to prevent/treat COVID-19. Quercetin is an acidic compound and shows metabolic interaction with some antivirals, antibiotics, and anti-inflammatory agents. Therefore, the physicochemical and metabolic drug interactions between quercetin and the combined drugs/compounds must be better understood before developing new compositions.

## 1. Introduction

At present, the world population is aware of the COVID-19 pandemic and its causative virus (SARS-CoV-2). The first case of COVID-19 was identified in December 2019, and the World Health Organization (WHO) declared it a global pandemic in March 2020 [1]. As of 4 March 2022, the WHO’s website shows 440,807,756 confirmed COVID-19 cases, and 5,978,096 deaths due to COVID-19, and 10,585,766,316 vaccine dosages administered to people [2]. The mild symptoms of COVID-19 include cough, fever, diarrhea, myalgia, fatigue, and anosmia. If untreated, it may lead to acute respiratory distress syndrome (ARDS), lung injury, multiple organ failures due to cytokine storm, thromboembolic diseases, and death [3]. Elderly people suffering from chronic diseases (diabetes, hypertension, etc.) and the immunocompromised population are particularly susceptible to SARS-CoV-2 infection [4]. Since the start of this pandemic, some antiviral drugs were reported to be effective against COVID-19, including remdesivir [5], molnupiravir [6], favipiravir [7], and monoclonal antibodies such as bamlanivimab [8]. Many vaccines were developed and marketed by Pfizer, Moderna, Johnson & Johnson, and Astra Zeneca for the prevention of COVID-19 [9]. Apart from drugs and vaccines, many food supplements (vitamins, minerals, and immune boosters) [10] and herbal products were claimed to prevent/treat COVID-19 [11]. Quercetin is one of the natural compounds that demonstrates its efficacy to prevent/treat COVID-19 alone, or in combination with other adjuvants/drugs. This fact is also supported by some published reviews on quercetin [12,13,14,15,16,17]. The present review highlights the prophylactic/therapeutic potential of quercetin against COVID-19, in view of clinical studies and patented compositions.

## 2. Searching for Non-Patent Literature

The keyword/combination keyword search with the terms “quercetin”, “quercetin + COVID-19”, and “quercetin + SARS-CoV-2” was performed on the PubMed and Scifinder databases. The appropriate non-patent literature related to general aspects of quercetin (Section 3), biological effects of quercetin (Section 3), the potential of quercetin to prevent/treat COVID-19 (Section 4), and the clinical studies of quercetin against COVID-19 (Section 5) were reviewed and incorporated in this review.

## 3. Quercetin

**Figure 1:**
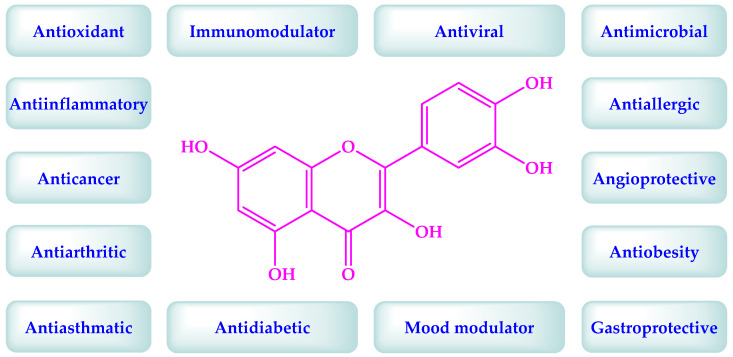
Chemical structure and biological effects of quercetin [18,21,22].

Quercetin (yellowish crystals; insoluble in water; soluble in acetic acid/alkali solutions; slightly soluble in alcohol; CAS registry number: 117-39-5; molecular weight: 302.24; melting point: 316.5 °C; chemical name: 2-(3,4-dihydroxyphenyl)-3,5,7-trihydroxy-4H-chromen-4-one) is a flavonol, which is found in many fruits (grape, apple, strawberry, cloudberry, and raspberry), and vegetables (onion, tomato, lettuce, and green bean) [18]. The chemical structure of quercetin is depicted in Figure 1. Quercetin contains four phenolic groups, which make it a strong scavenger of the reactive oxygen species (ROS), antioxidant, immune booster, and anti-inflammatory substance [18]. Quercetin is also an excellent metal ionophore (antioxidative, anti-inflammatory, and immunomodulatory zinc ionophore) [19], and also increases the glutathione (antioxidant) level in the body [20]. These properties make quercetin an important therapeutic agent to prevent/treat many diseases caused by oxidative stress and the release of pro-inflammatory substances in the body (Figure 1) [18,21,22].

**Figure 2:**
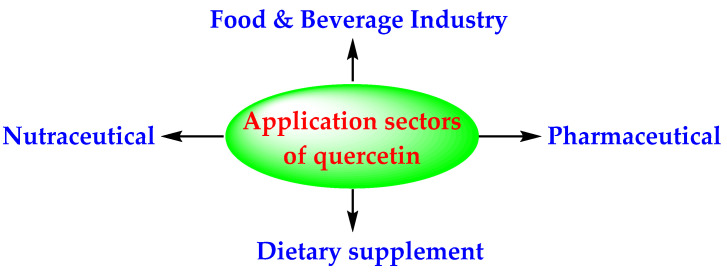
Application segments of quercetin according to end use [24].

Quercetin is obtained from the hydrolysis of rutin (a quercetin glycoside) [18]. The United States Food and Drug Administration (USFDA) designated quercetin as a GRAS (generally recognized as safe) substance, up to a level of 500 mg/day [23]. The releasable information (parts 1, 2, and 3) of the GRAS notice number 341 of the USFDA discusses the chemistry, manufacturing process, stability/photostability, impurities, metabolism, toxicology, clinical studies, and reason for granting the GRAS status to quercetin. In short, this states that the shelf life of pure quercetin is about 5 years; quercetin is absorbed from the stomach with 24–53% bioavailability; quercetin forms a albumin–quercetin complex that may be toxic in chronic use, but the limited bioavailability of quercetin prevents this event from happening; quercetin does not produce systemic toxicity up to 2.231 g/kg/day, or carcinogenicity; quercetin does not adversely impact the CYP450 enzymes and does not deplete the glutathione enzyme; quercetin is metabolized to two non-carcinogenic compounds, namely, 3′-O-methylquercetin and 4′-O-methylquercetin, and is excreted via urine as well as feces. Being a GRAS product, quercetin is used in nutraceuticals, the food/beverage industry, and as a diet supplement (Figure 2) [24].

## 4. Mechanism of Action of Quercetin against SARS-CoV-2

**Figure 3:**
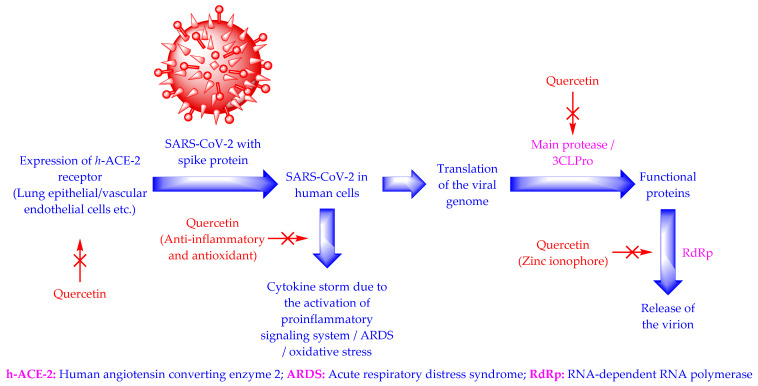
Life cycle of SARS-CoV-2 and mechanism of action of quercetin [5,6,9,12,25,26,27,28,29].

The SARS-CoV-2 virus is an RNA virus. It enters the human cell through the angiotensin-converting enzyme 2 receptors (hACE-2) [5,6,9]. The virus’s entry into the human cell causes the release of pro-inflammatory substances, oxidative stress, and acute respiratory distress syndrome [25,26]. The virus needs 3CLPro (main protease enzyme) and RNA-dependent RNA polymerase (RdRp) for its replication [5,6,9]. Quercetin demonstrates anti-COVID-19/anti-SARS-CoV-2 activity by lowering/inhibiting the expression of the hACE-2, the release of pro-inflammatory substances, 3CLPro, and RdRp [12,27,28,29] (Figure 3).

The molecular docking studies displayed the anti-SARS-CoV-2 activities of quercetin [12,27,28]. In silico studies using SARS-CoV-2 protease protein (PDB ID: 6LU7) demonstrate that quercetin inhibits SARS-CoV-2 protease by making hydrogen bonds with some residues (His164, Glu166, Asp187, Gln192, and Thr190) of 6LU7 [12,27]. Quercetin also reduces the expression of many human genes that encode SARS-CoV-2 proteins, and reduces the severity of SARS-CoV-2 infection [27]. It is known that the first phase of COVID-19 relates to immunodeficiency and the second phase is connected to a cytokine storm due to the overexpression of pro-inflammatory substances, including inflammasomes, cytokines, interleukin 1β, and interleukin 18 [27,28]. Therefore, immune boosters and anti-inflammatory therapies are recommended during the first and second phases of COVID-19, respectively [27]. Quercetin is a recognized antioxidant possessing immunomodulatory activity, anti-inflammatory activity, and anti-SARS-CoV-2 activity (Figure 1). Therefore, quercetin is believed to possess all the attributes required for COVID-19 therapy [12,18,21,22,27,28,29].

## 5. Clinical Studies of Quercetin against COVID-19

The clinical studies of quercetin were searched on the clinical trial database [30] on 8 March 2022, using the term “quercetin”. This search revealed 101 clinical studies related to the use of quercetin to prevent/treat various diseases, including oxidative stress, inflammation, viral infections (COVID-19), COPD, asthma, cardiovascular diseases (coronary artery disease/progression, atherosclerosis, endothelial dysfunction, etc.), carcinoma, aging, Fanconi anemia, and Alzheimer’s disease. This search also provided 15 clinical studies related to the use of quercetin, or its compositions, against COVID-19, which are summarized in Table 1. Among the 15 clinical studies, 11 were related to the treatment of COVID-19, 3 were for the prevention of COVID-19, and 1 was an observational study. Four studies have been conducted in Pakistan, two in Tunisia, and one each in Turkey, Saudi Arabia, the United States, Belgium, Italy, France, and Indonesia. Two studies did not provide the location of their studies. The clinical trials of Table 1 are related to the monotherapy of quercetin and isoquercetin, and their compositions including one or more substances selected from the group of zinc, vitamin C, curcumin, vitamin D3, masitinib, hydroxychloroquine, azithromycin, and ivermectin.

It is interesting to note that many clinical studies mentioned in Table 1 are not publicized as per the information provided in the database. Moreover, our search revealed more clinical studies relate to the efficacy of quercetin compositions against COVID-19. 

In one study, the combination of quercetin (1 g of quercetin daily for 7 days) with remdesivir and favipiravir is evaluated among hospitalized 60 COVID-19 patients. The intervention group patients (quercetin + remdesivir/favipiravir) have a better recovery rate than the control group patients (remdesivir/favipiravir). The quercetin-treated patients also show an improvement in hemoglobin level, respiratory rate, and ALP/LDH levels. The authors of this study recommend further investigations of the tested combination among ICU patients [31].

In another study, the administration of 1 dose/day oral composition comprising quercetin (400 mg), zinc (25 mg), quina (10 drops), vitamin C (1 g), vitamin D3 (25 mg), vitamin E (400 IU), and l-lysine (500 mg) among >100 individuals confirms the efficacy of the tested combination for the prophylaxis of COVID-19 [32].

One group of investigators disclose quercetin phytosome (a bioavailable phospholipid delivery form of quercetin) as possessing anti-SARS-CoV-2 activity [33]. They conducted a 2 week trial utilizing 21 outpatients availing standard care, and 21 outpatients availing standard care, in the combination of quercetin phytosome tablets (200 mg, 3 times/day). The results show that the incorporation of quercetin phytosome into standard care shortened the recovery time and severity of COVID-19. The quercetin phytosome-treated outpatients also show an improvement in the blood parameters (LDH, Ferritin, CRP, and D-dimer). As per the disclosure of this article, NCT04861298 (Table 1) is the registered clinical trial number of this study.

As per the literature, the study related to NCT04578158 (Table 1) has also been published [34]. This study also relates to the quercetin phytosome. The quercetin phytosome (1 g/day) was given to 153 COVID-19 outpatients. The results are promising and demonstrate a decrease in the recovery time, oxygen demand, and hospitalization need. The investigators also state quercetin phytosome is an anti-fatigue and pro-appetite composition with a good safety profile.

The published result of NCT05037240 (Table 1) [35] relates to quercetin phytosome (250 mg 2 times/day) among 120 subjects and indicates that the co-administration of quercetin phytosome with standard care provides more protective effects against COVID-19 than a placebo. In addition, further studies are recommended to use quercetin as a general prophylactic of SARS-CoV-2 infection. 

The results of NCT04377789 (Table 1) have also been publicized [35]. This study was conducted on 429 patients utilizing a composition comprising quercetin, vitamin C, and bromelain in combination with standard care. The administered composition improves the recovery rate and the blood parameters also. Moreover, the article recommends further studies to assess the dose/response relationship and the bioavailability of the quercetin composition.

The Shufeng Jiedu capsules comprising polydatin, wogonin, and quercetin as its main components were evaluated on 43 patients as a possible treatment of COVID-19 [36]. The addition of the capsule with the standard antiviral therapy significantly decreases the recovery time and the symptoms (fatigue and cough) of COVID-19 patients. The investigators also suggest a large-scale clinical trial related to the subject matter.

A clinical trial on 97 patients was reported for Yindan Jiedu granules [37]. The main components of these granules are luteolin, quercetin, and kaempferol. The results demonstrate the promising anti-inflammatory effects of Yindan Jiedu granules on COVID-19 patients.

## 6. Patent Searching

The patent search was performed on 8 March 2022, employing different patent databases described in the literature [38,39,40,41,42], using the combination terms “Quercetin + COVID-19” and “Quercetin + SARS-CoV-2”. The patent database, Espacenet (quercetin + COVID-19 = 45 hits and quercetin + SARS-CoV-2 = 47 hits), Sci-Finder (quercetin + COVID-19 = 47 hits and quercetin + SARS-CoV-2 = 18 hits), USPTO (quercetin + COVID-19 = 31 hits and quercetin + SARS-CoV-2 = 29 hits), and Patentscope (quercetin + COVID-19 = 44 hits and quercetin + SARS-CoV-2 = 45 hits) revealed many patents and patent applications (Table 2). The duplicate patent family members were removed. The patents/patent applications of a single patent family that specifically mentioned quercetin, or its compositions, in the claim section for the prevention and treatment of COVID-19 were included for further analysis. The patent applications claiming the use of novel non-clinical (clinical studies have never been performed) compounds in combination with quercetin were excluded. The patent/patent applications containing a broad dependent claim concerning quercetin without any experimental support were also excluded. The information about the international patent classification (IPC), priority countries, publication date, and the status of the cited patents/patent applications were obtained from the Espacenet and USPTO databases.

It is evident from the data of Table 2 that only a few patent applications provide examples related to the use of quercetin, or its compositions, to prevent/treat COVID-19. Other patent applications claim the use of quercetin as an optional ingredient of the claimed composition. Accordingly, an analysis of the patent applications that contain examples of using quercetin or its compositions to prevent/treat COVID-19 is provided below.

**US2021315857A1** covers a pharmaceutical micronutrient composition comprising ascorbate (10 mg to 200,000 mg), *N*-acetylcysteine (2 mg to 30,000 mg), theaflavin (5 mg to 3000 mg), resveratrol (10 mg to 5000 mg), cruciferous plant extracts (5 mg to 5000 mg), curcumin (5 mg to 10,000 mg), quercetin (5 mg to 2000 mg), naringenin (5 mg to 3000 mg), polyphenol extract from green tea (1 mg to 10,000 mg), brazilin (1 mg to 5000 mg), and baicalin (5 mg to 3000 mg) [43]. The claimed composition inhibits the SARS-CoV-2 entry into the body by decreasing the expression of hACE-2 receptors. The in vitro study reveals that the composition D (resveratrol, cruciferous plant extract, curcumin, quercetin, naringenin, baicalin, theaflavin, vitamin C, and *N*-acetylcysteine) provides the best inhibition of receptor binding of SARS-CoV-2 to hACE-2. However, the patent application is silent about the rationale of quercetin in the claimed composition.

**WO2021259441A1** provides a composition containing quercetin and tamarixin [44]. The patent application states that quercetin and tamarixin bind with ACE2, and prevent the binding of SARS-CoV-2 with the ACE2; they also inhibit the formation of ACE2 from ACE1, which results in several beneficial events (vasodilatation, vascular protection, antifibrosis, antiproliferation, antiinflammation, and increase nitric oxide release), and act as a metal ionophore for zinc, which has antiviral effects. The clinical trial studies provided in the specification of this patent application reveal that the claimed composition has a prophylactic effect against COVID-19; improves rhinitis, pharyngitis, and general symptoms of fatigue; decreases the need for the antibiotic duration; prevents acute respiratory distress syndrome (ARDS); protects lungs from infection/complications of COVID-19; can be used as an add on treatment for moderate cases infected with coronaviruses; and can be used as an antifibrotic drug for the treatment of interstitial pulmonary fibrosis.

**WO2021257252A1** claims a composition encompassing luteolin (80 mg to 220 mg), quercetin (90 mg to 310 mg), kaempferol (80 mg to 220), vitamin C (475 mg to 525 mg), and a pharmaceutically acceptable carrier [45]. The claimed composition utilized quercetin as an inhibitor of the expression of the hACE-2 receptors, and luteolin as a furin inhibitor. It is claimed that both of these components reduce the pathogenicity of the SARS-CoV-2 virus. The presence of kaempferol and vitamin C in the composition enhances the bioavailability of luteolin and quercetin. The clinical studies data of the claimed invention reveal improvement in COVID-19 symptoms among patients after 1 day of the treatment, and the symptoms disappeared completely after 3 days.

**WO2021255464A1** claims a nutraceutical composition comprising curcumin (1 to 8%), vitamin C (40 to 90%), chlorella (5 to 20%), spirulina, and Boswellia resin (1.0 to 15) that may optionally contain quercetin (0.5 to 5%) [46]. The clinical data provided in the patent application suggests that the claimed composition improves the COVID-19 symptoms among the patient. The composition utilizes quercetin as an antioxidant, antiviral, and anti-inflammatory compound. However, the clinical data are silent about the exact role of quercetin.

**WO2021240481A2** claims an oral composition comprising hesperidin (10% to 25%), curcumin (15% to 35%), epigallocatechin (15% to 30%), rutin (10% to 25%), quercetin (0.5% to 8%), luteolin (1% to 10%), baicalin (1% to 15%), piperine (0.03% to 3%), and one or more excipients [47]. The claimed composition exhibits functional synergy among its ingredients; prevents virus replication and/or virus entry into human cells; modulates the immune response of the patient, and protects organs/cells. However, this patent application is silent about the functional role/rationale of using quercetin in the composition.

**WO2021205083A2** relates to the composition containing 2,6-di-tert-butyl-4-methyl-phenol (BHT, 0.01 mg to 1 g) alone, or in combination with another antioxidant (quercetin, 100 to 200 mg) and/or an anti-inflammatory agent (*N*-acetylcysteine, 100 mg) possessing antiviral activity against SARS-CoV-2 [48]. The patent application provides in vitro data and clinical protocols related to the use of BHT and its compositions against SARS-CoV-2 infected cells. However, the patent application is published in French. The English translation of the text on Espacenet is not well defined. Therefore, the authors avoid discussing the experimental results due to a lack of clarity.

**WO2021168173A1** claims a nasal composition of quercetin (0.5% to 25% *w*/*w*) and iota-carrageenan (0.05 to 5.0% *w*/*w*), along with other ingredients, including ethyl lactate [49]. It demonstrates that ethyl lactate increases the solubility of quercetin 10-fold. The examples demonstrate that the DMSO solution of quercetin (140 uM) inhibits the main protease (Mpro) of SARS-CoV-2 by about 95%, in comparison to disulfiram (a known protease inhibitor). Another example shows that iota-carrageenan treatment reduces the proportion of SARS-CoV-2 infected Vero cells in a dose-dependent manner. The third example verifies that the combination of quercetin and iota-carrageenan in different concentrations also reduces the number and the ratio of SARS-CoV-2 infected A549 cells. The composition is supposed to inhibit the viral entry into the cells by lowering the expression of hACE-2 receptors.

**US2022040227A1** claims a composition comprised of copper chelator (tetra-thiomolybdate salt), a 5-lipoxygenase inhibitor (diethylcarbamazine or Zileuton), and quercetin [50]. This invention recognizes that the COVID-19 can become lethal because of its inflammatory damage to the lung/heart. The claimed invention treats COVID-19 because of its anti-inflammatory effects. This patent application is silent about the experimental data of the claimed invention against COVID-19.

**US2021393579A1** claims a composition (sugar syrup) comprising anthocyanins (extracted from a food plant) and quercetin [51]. The composition uses a sugar-based agent (sucrose) to obtain the quercetin into a solution and to facilitate the absorption of quercetin in the body. The composition claims to improve the respiratory/joint health of the patient by reducing the inflammation of the lung cells/joints with the help of prophetic examples.

**US2021386779A1** claims a composition containing a zinc ionophore (quercetin, 50 mg to 1500 mg) and a bio-assimilable form of zinc (Zn^+2^) (5 mg to 100 mg) to enhance the immune system function [52]. This dietary supplement may optionally contain l-lysine, vitamin C, vitamin D, and vitamin E. The clinical study data (5 weeks and 20 weeks) strongly suggests that zinc supplementation (25 mg daily) together with zinc ionophores (Quina tree bark extract and quercetin), along with vitamins C/D3/E, and l-lysine provides a strong protective prophylactic effect against COVID-19.

**US2021361700A1** claims a composition encompassing at least two components selected from the group consisting of zinc (10–75 mg/day), quercetin (100–1500 mg/day), vitamin E (50–800 mg/day), and epigallocatechin gallate (EGCG, 300–600 mg/day) [53]. All four components of the composition were selected based on their antiviral effects against COVID-19. The patent application provides a prophetic clinical protocol to study the effect of the composition against COVID-19. The invention claims to reduce the hospitalization time, intubation time, disease duration, limit the clinical progression, and reduce the symptom severity of COVID-19. The composition also inhibits viral entry into cells and viral reproduction in the cell; increases T cell function; reduces inflammation, cytokine levels, reactive oxygen species, and tissue damage in the subject.

**US2021290718A1** claims a composition (oral aerosolized spray) comprising quercetin, vitamin C (250 mg to 10,000 mg), vitamin D (1000 mg to 100,000 mg), zinc (5 mg to 100 mg), and artemisia to prevent/treat COVID-19 [54]. This is interesting to note that this patent application provides clinical studies related to the compositions of hydroxychloroquine, azithromycin, vitamin C, vitamin D, and zinc. However, it is silent about the rationale of quercetin and its concentration in the claimed composition.

**CN112457281A** provides quercetin disulfonated derivatives that block the binding of COVID-19 spike protein to the human angiotensin-converting enzyme 2 (hACE-2) [55]. One of the quercetin disulfonated derivatives, at a concentration of 7.6 μM, demonstrates 51.8% inhibition of the COVID-19 spike protein receptor-binding domain and Hela cells expressing hACE-2. The higher concentration (>250μM) also demonstrates more than 75% inhibition.

**CN112263598A** covers the aqueous ethanol extract of Tianjihuang [56]. The in vitro antiviral activity against SARS-CoV-2 infected VeroE6 cells demonstrate better antiviral activity of the Tianjihuang extract (IC_50_ = 12.70 µg·mL^−1^) than quercetin (IC_50_ = 52.24 µg·mL^−1^) and proleucoside B (IC_50_ = 32.84 µg·mL^−1^). However, no mechanism of action is postulated for the antiviral effect of the extract. The higher antiviral activity of the extract is attributed to its chemical constituents (quercetin, isoquercetin, quercetin B, quercetin, etc.).

**CN112022845A** claims the use of quercetin (dose 12.5 to 100 μg/mL) to treat COVID-19 [57]. The experiments reveal a reduction in the SARS-CoV-2 viral load in virus-infected Vero cells after 48 h of the administration of the DMSO solution of quercetin (2.86, 4.07, 23.31, and 1290 times reduction after the 12.5, 25, 50, and 100 μg/mL dose of quercetin), in comparison to non-treated virus-infected cells. The inventors identify that the dose of quercetin (>100 μg/mL) is cytotoxic for Vero cells. Accordingly, they suggest a dose of quercetin < 100 μg/mL to treat COVID-19. However, this document is silent about the mechanism of action of quercetin.

**WO2021262749A1** claims a pharmaceutical composition comprising quercetin (0.1 g to 2.5 g), and hydrogen peroxide (1–3 mL) [58]. The claimed composition shows a surprising and unexpected antiviral effect against SARS-CoV-2. The composition further demonstrates improved results among cancer patients suffering from COVID-19, by inhibiting the PI3K gene. The clinical data provided in the specifications of this patent application supports the efficacy of the claimed invention against COVID-19, as well as among cancer patients sufferings from COVID-19.

## 7. Conclusions

The clinical trials, inventive compositions, and the patent literature on quercetin demonstrate its potential as a prophylactic/therapeutic agent against COVID-19, because of its inhibitory effect on the expression of the human ACE2 receptors, enzymes of SARS-CoV-2 (MPro, PLPro, and RdRp), antioxidant activity, anti-inflammatory activity, and immunomodulatory activity. Quercetin is a GRAS substance, as per the USFDA. Accordingly, it may be used in combination with clinically used antivirals, and drugs that control the symptoms of COVID-19. Therefore, the authors imagine the development of many more quercetin-based inventive/patentable compositions as monotherapy or combination therapy for the prevention/treatment of COVID-19. However, the potential interactions between the combined drugs must be understood before developing a new composition.

## 8. Discussion

Quercetin is an established antioxidant and anti-inflammatory compound [18]. It demonstrates its anti-COVID-19 activity by inhibiting the expression of ACE2 receptor, and enzymes of SARS-CoV-2 (3CLPro and RdRp) (Figure 1). These facts were confirmed based on in silico, in vitro, in vivo, and clinical studies (Table 1 and Table 2) [12,14]. The infection of SARS-CoV-2 can also cause cytokine storms. If untreated, the cytokine storm leads to blood clotting, multiple organ failure, and ultimately death (Figure 1) [3]. Quercetin can prevent the trigger of a cytokine storm, and blood clotting, due to its antioxidant, anti-inflammatory, and anti-thrombin activity [12]. The clinical studies on quercetin-based monotherapy and combination therapy with zinc, vitamin C, curcumin, vitamin D3, masitinib, hydroxychloroquine, azithromycin, and ivermectin were promising against COVID-19 (Table 1) [31,32,33]. These clinical studies demonstrate faster recovery and a decrease in the hospitalization time of the COVID-19 patients. Quercetin is also in a clinical trial for diseases such as oxidative stress, inflammation, cardiovascular diseases, and carcinoma [30]. Therefore, the compositions containing quercetin may be useful to treat COVID-19 among the high-risk population (hypertensive, diabetics, cancer, etc.). The initial SARS-CoV-2 infection does not need advanced treatment. Quercetin is present in many fruits, vegetables, and health supplements. The studies also advocate a regular intake of quercetin-containing food (onion, etc.) and quercetin supplements during the pandemic [14]. However, it is still questionable if quercetin-rich food/supplements can be used prophylactically for COVID-19 among the high-risk population. Quercetin is lipophilic and its low water solubility limits its absorption [16]. This led to the discovery of a phospholipid-based delivery system of quercetin with improved bioavailability, which displays promising outcomes to treat/prevent COVID-19 [33]. This discovery opens the doors to developing more anti-COVID-19 quercetin conjugates, prodrugs, liposomes, nano-formulations, and nano-particle systems possessing a better pharmacokinetic profile.

**Figure 4:**
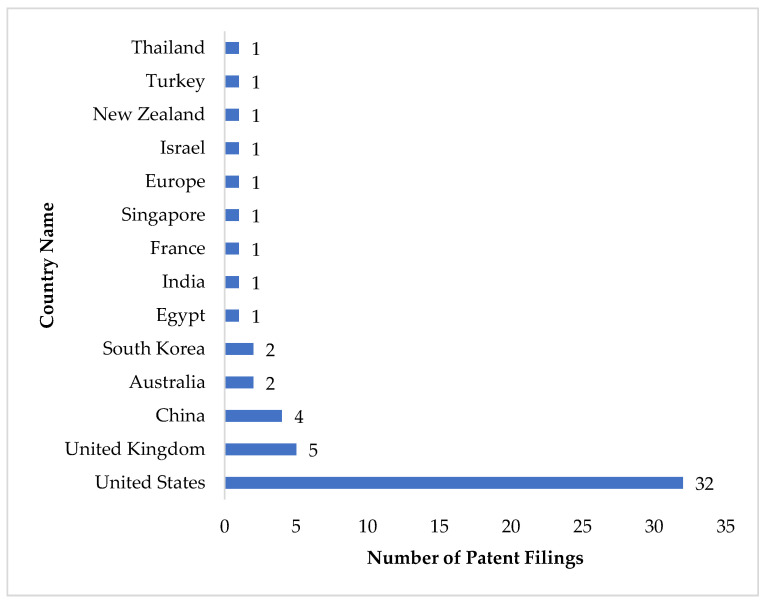
The patent application filing countries related to the quercetin-based anti-COVID-19 compositions.

The authors searched the patent literature on quercetin and COVID-19. Most of the patent applications were filed in the United States (Figure 4). The majority of these patent applications were published in 2021, which is expected. Many of these patent applications provide evidence (in silico, in vitro, in vivo, and clinical studies) for the prophylactic/therapeutic potential of quercetin and its nutraceutical/pharmaceutical compositions (oral and inhale formulations) against COVID-19 (Table 2).

**Figure 5:**
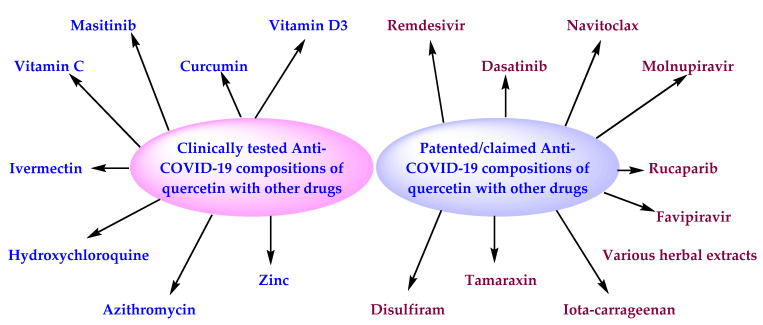
Important anti-COVID-19 combinations of quercetin [44,45,46,47,48,49,50,51,52,53,54,55,56,57,58,59,60,61,62,63,64,65,66,67,68,69,70,71,72,73,74,75,76,77,78,79,80,81,82,83,84,85,86,87,88,89,90,91,92,93,94,95,96], Table 2.

The anti-SARS-CoV-2 effect of quercetin in specific doses (12.5 to 100 μg/mL) is claimed in a Chinese patent application [57]. This patent application filing also strengthens the concept of utilizing quercetin as an anti-COVID-19 agent. The combinations of quercetin with favipiravir, remdesivir, molnupiravir, navitoclax, dasatinib, disulfiram, rucaparib, tamarixin, iota-carrageenan, and various herbal extracts (aloe, poria, rosemary, and sphagnum) are also claimed in patent applications (Table 2 and Figure 5) [43,44,45,46,47,48,49,50,51,52,53,58]. Most of these combinations were developed as an oral dosage form (tablet, solution, capsule, oral inhaler, etc.), and reveal synergistic/additive anti-SARS-CoV-2 effects. It is quite interesting to note that the patent applications claim combinations of quercetin with a large number of antioxidants, antivirals, immunomodulators, and anti-inflammatory agents. However, these patent applications exemplify the anti-SARS-CoV-2 effect of only a few combinations (Table 2). This indicates that there are a large number of experimentally unexplored quercetin combinations. Therefore, there is a high possibility that some of the experimentally unexplored quercetin combinations may provide unexpected/surprising results against COVID-19.

**Figure 6:**
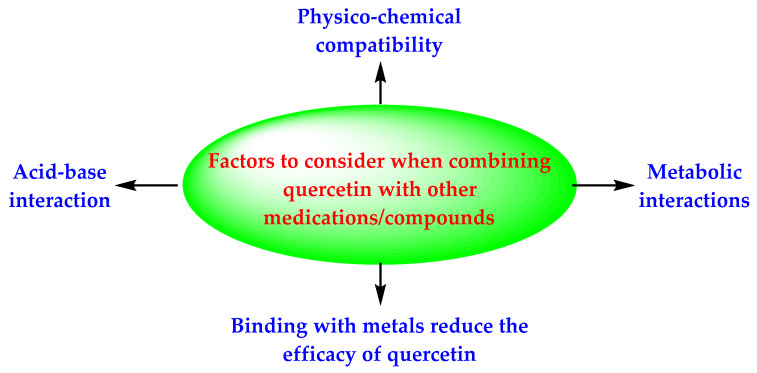
Factor that may affect the quercetin-based drug combinations.

It is important to note that quercetin is an acidic compound [18], due to the presence of phenolic groups in its structure (Figure 1). Therefore, it shows the acid–base interaction with basic drugs/compounds (Figure 6). This interaction affects the biological response of quercetin. Quercetin is also a GRAS substance, according to the USFDA., but it can alter the metabolism of some drugs [18]. The literature has highlighted interactions of quercetin with many drugs, including ivermectin, antibiotics (azithromycin, ciprofloxacin, ofloxacin, itraconazole, and posaconazole), antivirals (indinavir, nelfinavir, ritonavir, and saquinavir), and steroidal anti-inflammatory drugs (cortisone and dexamethasone). It is known that the treatment of COVID-19 encompasses antivirals, antibiotics, and anti-inflammatory agents. Accordingly, the authors trust that the physicochemical and metabolic drug interactions of quercetin must be understood before making a combination of quercetin with other drugs/compounds. Further, quercetin is also an established metal ionophore [19]. It binds with beneficial metals like zinc, which provides a synergistic effect. However, the binding of quercetin with non-beneficial metals should also be considered while making combinations.

To date, many variants of COVID-19 (α, β, γ, δ, Omicron, etc.) with a high transmission rate were identified [2]. The appearance of these variants is posing challenges to the existing vaccines and drugs (molnupiravir and remdesivir) used against COVID-19 [2,43]. A multicomponent composition comprising quercetin that is effective against coronavirus mutants is reported [44]. However, the specific use/effect of quercetin against coronavirus mutants is not clarified in this report [43]. The emergence of many variants of COVID-19 makes it essential to develop safe and inexpensive treatments for COVID-19. Quercetin is a GRAS substance, inexpensive, and demonstrates beneficial effects among the high-risk population. The authors foresee quercetin as a potential candidate to develop an anti-COVID-19 therapy. Therefore, quercetin, and its drug combinations, must be explored clinically on a large scale to identify a potential quercetin-based COVID-19 treatment. The clinical efficacy of quercetin against emerging variants of COVID-19 (α, β, γ, δ, Omicron, etc.) also needs further investigation.

## Figures and Tables

**Table 1:** Clinical studies on quercetin and its compositions against COVID-19.

Intervention	Primary Purpose (Phase; Status; Results)	NCT Number (Completion Date)	Sponsor (Location of the Clinical Trial)
Standard care + Quercetin phytosome (400 mg/day for 30 days)	Treatment (3; completed; published)	NCT04578158 (15 April 2021)	Liaquat University of Medical & Health Sciences (Pakistan)
Quercetin (500 mg/day) for non-COVID-19 individual group for 3 months	Prevention (not mentioned; completed; published)	NCT04377789 (31 August 2020)	Kanuni Sultan Suleyman Training and Research Hospital (Turkey)
Standard care + 600 mg/day of quercetin for the first week and 400 mg/day of quercetin for the second week	Treatment (not mentioned; completed; published)	NCT04861298 (29 August 2021)	King Edward Medical University Teaching Hospital (Pakistan)
Dietary supplement (zinc and vitamin C) + quercetin (500 mg/day) to COVID-19 patients for 4 weeks	Treatment (4; recruiting; not posted)	NCT04468139 (30 July 2020)	Ministry of Health, First Health Cluster (Saudi Arabia)
Standard care + a combination of quercetin and curcumin (No dose mentioned)	Treatment (not mentioned; completed; not posted)	NCT05130671 (31 December 2021)	King Edward Medical University (Pakistan)
Standard care + quercetin (268 mg/day) + curcumin (168 mg/day) + vitamin D3 (360 IU) for 14 days	Treatment (not mentioned; recruiting; not posted)	NCT05008003 (31 March 2022)	Liaquat University of Medical & Health Sciences (Pakistan)
Quercetin (500 mg, 2 times/day) for 3 months	Prevention (not mentioned; completed; published)	NCT05037240 (25 May 2021)	Personal Services Company of Pavia (Italy)
Quercetin tablet 3 times/day (dose not mentioned) for 10 days before the meal	Treatment (1; completed; not posted)	NCT04851821 (30 June 2021)	Sahloul University Hospital (Tunisia)
Masitinib (3 mg/kg/day for 4 days, then 4.5 mg/kg/day) + isoquercetin (prodrug of quercetin, 1 g/day orally) for 15 days	Treatment (2; recruiting; not posted)	NCT04622865 (June 2022)	AB Science (France)
Two quercetin tablets (dose not mentioned) per day before meal for 10–30 days	Treatment (1; recruiting; not posted)	NCT04853199 (30 August 2021)	Sahloul University Hospital (Tunisia)
Standard care (zinc and vitamin C) + 2 capsules of the extract of Psidii guava (a source of quercetin) 3 times a day for 1 week	Treatment (3; completed; not posted)	NCT04810728 (30 January 2021)	Baiturrahmah University (Indonesia)
Hydroxychloroquine (0–400 mg) + azithromycin (0–500 mg + dietary supplements, including 0–600 mg quercetin (exact doses not mentioned)	Prevention (1; withdrawn; not posted)	NCT04590274 (December 2021)	International Brain Research Foundation (Not mentioned)
Isoquercetin (prodrug of quercetin) 1 g 2 times/day on day 1, followed by 500 mg 2 times/day for 27 days	Treatment (2; not yet recruiting; not posted)	NCT04536090 (June 2023)	Montreal Clinical Research Institute (Not mentioned)
Ivermectin (0.4 mg/kg) to treat outpatients, including supplemental treatment comprising quercetin (dose not mentioned)	Observational (not mentioned; not yet recruiting; not posted)	NCT05045937 (20 September 2023)	Patrick Robinson (United States)
NASAFYTOL (capsules encompassing quercetin, turmeric extract, and vitamin D3) for 14 days	Treatment (not mentioned; completed; not posted)	NCT04844658 (29 October 2021)	Tilman and Artialis (Belgium)

**Table 2:** The data and the summary of the claimed invention in patent/patent applications.

Patent/Application Number (Applicant; Publication Date; Priority Country)	Summary of the Claimed Invention to Prevent/Treat COVID-19	Examples of Studies on Quercetin or Its Compositions
**US2021315857A1** (Rath Matthias W.; 14 October 2021; United States)	A composition comprising many ingredients, including quercetin [43]	In vitro
**WO2021259441A1** (Khalil et al.; 30 December 2021; Egypt)	A composition containing quercetin and tamarixin [44]	Clinical
**WO2021257252A1** (Hofleitner Peter; 23 December 2021; United States)	A composition encompassing luteolin, quercetin, kaempferol, vitamin C [45]	Clinical
**WO2021255464A1** (Hahn Norman; 23 December 2021; United Kingdom)	A nutraceutical composition comprising many ingredients, including quercetin [46]	Clinical
**WO2021240481A2** (Vedicinals India Private Limited; 2 December 2021; India)	A composition comprising many active ingredients, including quercetin [47]	In vitro and clinical
**WO2021205083A2** (Spadavecchia et al.; 14 October 2021; France)	A composition of 2,6-di-tert-butyl-4-methyl-phenol that may optionally contain quercetin [48]	In vitro
**WO2021168173A1** (Synkine Therapeutics; 26 August 2021; United States)	A composition of quercetin and iota-carrageenan [49]	In vitro
**US2022040227A1** (ReversPAH; 10 February 2022; United States)	A composition comprising a copper chelator, a 5-lipoxygenase inhibitor, and quercetin [50]	Not present
**US2021393579A1** (Zestt Wellness Limited; 23 December 2021; United States)	A composition comprising anthocyanins and quercetin [51]	Prophetic clinical study
**US2021386779A1** (Margolin Leon; 16 December 2021; United States)	A composition containing a zinc ionophore (quercetin) and a bio-assimilable form of zinc (Zn^+2^) [52]	Clinical
**US2021361700A1** (Tufts College; 25 November 2021; United States)	A composition encompassing zinc, quercetin, vitamin E, and epigallocatechin gallate [53]	Prophetic clinical study
**US2021290718A1** (Hazan Sabine; 23 September 2021; United States)	A composition comprising quercetin, vitamin C, vitamin D, zinc, and artemisia [54]	Clinical
**CN112457281A** (Dalian University of Technology; 9 March 2021; China)	Quercetin disulfonated derivatives [55]	In vitro
**CN112263598A** (Sun Yat-Sen University; 26 January 2021; China)	Aqueous ethanol extract of Tianjihuang containing quercetin [56]	In vitro
**CN112022845A** (Zhejiang Provincial Center for Disease Control and Prevention; 4 December 2020; China)	Use of quercetin (dose 12.5 to 100 μg/mL) to treat COVID-19 [57]	In vitro
**WO2021262749A1** (Research Cancer Institute of America; 30 December 2021; United States)	A composition consisting of quercetin and hydrogen peroxide [58]	Clinical
**US10729735B1** (Phoenix Biotechnology; 4 August 2020; United States)	A composition comprising oleandrin that may optionally contain quercetin [59]	Not present
**WO2022024097A1** (AI Pharmaceuticals; 3 February 2022; United States)	A composition comprising edible mushrooms that may optionally contain quercetin [60]	Not present
**WO2022019828A1** (National University of Singapore; 27 January 2022; Singapore)	A composition comprising mast cell stabilizers, including quercetin [61]	Not present
**US2022016196A1** (Unigen; 20 January 2022; United States)	A composition containing aloe extract, poria extract, and rosemary extract [62]	Not present
**WO2022015570A1** (University of California; 20 January 2022; United States)	A composition comprising one or more mitochondrial antioxidants, including quercetin [63]	Not present
**US20220001014A1** (Ghaderi Lida; 6 January 2022; United States)	A composition comprising an inflammation/protease/cytokine inhibitors, and angiotensin receptor 1 inhibitor (quercetin) [64]	Not present
**WO2021248254A2** (Ingenew Pharma; 16 December 2021; United States)	A composition including a flavonoid (hesperidin, hesperetin, naringin, naringenin, diosmin, quercetin, and myricetin) [65]	Not present
**WO2021245365A1** (Remedy Research Limited; 9 December 2021; United Kingdom)	An aqueous composition comprising zinc ions or zinc ionophores (quercetin), sulfur ions, and ammonium ions [66]	Not present
**WO2021242590A1** (Phoenix Biotechnology; 2 December 2021; United States)	A composition encompassing oleandrin and another compound (quercetin) [67]	Not present
**WO2021237292A1** (Genetic Technologies Limited; 2 December 2021; Australia)	A method of treating COVID-19 using drugs (favipiravir, remdesivir, quercetin, etc.) [68]	Not present
**WO2021233948A1** (INSERM; 25 November 2021; Europe)	A composition of senotherapeutic compounds (navitoclax, dasatinib, quercetin, etc.) [69]	Not present
**WO2021237215A1** (University of Louisville Research Foundation; 25 November 2021; United States)	A composition comprising plant-derived (curcumin, resveratrol, quercetin, etc.) exosome-like particles [70]	Not present
**WO2021234362A1** (University of Wolverhampton; 25 November 2021; United Kingdom)	A non-orally administered composition encompassing disulfiram that may optionally contain quercetin [71]	Not present
**US20210353685A1** (Brain Cancer Research Institute; 18 November 2021; United States)	A method to increase the efficacy of the cell therapy using antioxidants (ascorbic acid, tocopherol, rutin, quercetin, etc.) [72]	Not present
**WO2021226627A1** (Figene; 11 November 2021; United States)	A composition of sphagnum extract that may optionally contain quercetin [73]	Not present
**WO2021222240A1** (Systamedic; 4 November 2021; United States)	A composition containing a polymerase inhibitor (rucaparib, fisetin, quercetin, etc.) and a viral replication inhibitor (remdesivir, molnupiravir, favipiravir, etc.) [74]	Not present
**WO2021220137A2** (Sapir Pharmaceuticals; 4 November 2021; United States)	A composition of pannexin-1 inhibitor (probenecid) that may optionally contain quercetin [75]	Not present
**WO2021216562A1** (Finzi Eric; 28 October 2021; United States)	A composition of zinc that may optionally include a zinc ionophore (quercetin) [76]	Not present
**WO2021216749A1** (Hoag George Edward and Salerno John; 28 October 2021; United States)	A composition comprising TRPA1 antagonists (1,8-cineole), a plant extract of antibacterial/antiviral compounds (b-caryophyllene, etc.), and a plant extract of antioxidants (quercetin, etc.) [77]	Not present
**WO2021209740A1** (Mereo Biopharma 4 Limited and UAB Research Foundation; 21 October 2021; United Kingdom)	A composition of alvelestat that may optionally include quercetin [78]	Not present
**WO2021205437A1** (Vecht-Lifshitz Susan Eve; 14 October 2021; Israel)	A composition comprising a methyltransferase inhibitor, a viral enzyme inhibitor, and a SAHH (s-adenosylhomocysteine hydrolase) inhibitor that may optionally contain quercetin [79]	Not present
**WO2021206566A1** (Manukamed Limited Partnership; 14 October 2021; New Zealand)	A composition of manuka honey that may optionally contain quercetin [80]	Not present
**WO2021202103A2** (Phoenix Biotechnology; 7 October 2021; United States)	A composition comprising oleandrin, and digoxin that may optionally include quercetin [81]	Not present
**WO2021201903A1** (Phoenix Biotechnology; 7 October 2021; United States)	A composition comprising oleandrin, and digoxin that may optionally include quercetin [82]	Not present
**WO2021202032A1** (Figene; 7 October 2021; United States)	A composition containing fibroblasts and/or fibroblast-derived exosomes, and immune regulatory cells that may optionally include NF-kappa B inhibitor (quercetin) [83]	Not present
**WO2021194436A1** (Nanobiomed Saglik Ve Yasam Bilimleri; 30 September 2021; Turkey)	A composition of quercetin, which is isolated from Papaver rhoeas red petals [84]	Not present
**WO2021186250A2** (Davidoff Allen; 23 September 2021; United States)	A composition comprising a uric acid lowering agent (uricase) that may optionally contain antioxidants (quercetin, etc.) [85]	Not present
**WO2021160982A1** (Nasaleze Patents; 19 August 2021; United Kingdom)	A composition including HPMC particles, and a signaling agent (menthol, strawberry, etc.) that may optionally contain a biologically active agent (quercetin) [86]	Not present
**AU2021106876A4** (Apex Biotech Research; 25 November 2021; Australia)	A composition of andrographolide, ursolic acid, and piceid that may optionally contain an antiviral compound (quercetin) [87]	Not present
**WO2021211620A1** (Sun Genomics; 21 October 2021; United States)	A composition comprising a probiotic, prebiotic, and/or metabolite of the gut microbiome that may optionally contain an antiviral (quercetin) [88]	Not present
**US2021293827A1** (Winchester Henry; 23 September 2021; United States)	A composition of an anti-inflammatory drug, an anticoagulant, a thrombolytic, an immunosuppressive agent, a metal chelator, an iron supplement, or a nutraceutical (quercetin) [89]	Not present
**KR20210141341A** (APRG Company; 23 November 2021; South Korea)	A composition comprising ursolic acid or quercetin isolated from Yongacho [90]	Not present
**WO2021230667A1** (Mecox Curemed and Choongang Ocean; 18 November 2021; South Korea)	A composition comprising Zanthoxylum piperitum leaf extract, or a compound isolated from it (quercetin of its analog) [91]	Not present
**CN111773282A** (Golden Health Foshan Technology Company; 16 October 2020; China)	A Chinese composition containing many natural ingredients, including quercetin [92]	Not present
**WO2021236023A1** (Rincome Udom; 25 November 2021; Thailand)	A fermented Plu kow leaf powder containing quercetin [93]	Not present
**US20220047545A1** (Rath Matthias; 17 February 2022; United States)	A composition containing many natural compounds including quercetin [94]	Present, but no comment on quercetin
**US20210308186A1** (Brain Cancer Research Institute; 7 October 2021; United States)	A composition of NF-kappa B activity blockers (quercetin, etc.) [95]	Not present
**US2021315910A1** (Stafford Vivi Robyn; 14 October 2021; United States)	A composition of doxycycline that may optionally contain quercetin [96]	Clinical

## References

[1] Pollard C.A., Morran M.P., Nestor-Kalinoski A.L. (2020). The COVID-19 pandemic: A global health crisis. Physiol. Genom..

[2] Coronavirus Disease (COVID-19) Pandemic. https://www.who.int/emergencies/diseases/novel-coronavirus-2019.

[3] Lane A., Hunter K., Lee E.L., Hyman D., Bross P., Alabd A., Betchen M., Terrigno V., Talwar S., Ricketti D. (2021). Clinical characteristics and symptom duration among outpatients with COVID-19. Am. J. Infect. Control.

[4] Yang Y., Zhao Y., Zhang F., Zhang L., Li L. (2020). COVID-19 in Elderly Adults: Clinical Features, Molecular Mechanisms, and Proposed Strategies. Aging Dis..

[5] Imran M., Alshrari A.S., Asdaq S.M.B., Abida (2021). Trends in the development of remdesivir based inventions against COVID-19 and other disorders: A patent review. J. Infect. Public Health.

[6] Imran M., Kumar A.M., Asdaq S.M.B., Khan S.A., Alaqel S.I., Alshammari M.K., Alshehri M.M., Alshrari A.S., Mateq A.A., Al-Shammeri A.M. (2021). Discovery, Development, and Patent Trends on Molnupiravir: A Prospective Oral Treatment for COVID-19. Molecules.

[7] Hashemian S.M., Farhadi T., Velayati A.A. (2021). A review on favipiravir: The properties, function, and usefulness to treat COVID-19. Expert Rev. Anti. Infect. Ther..

[8] Asdaq S.M.B., Rabbani S.I., Alkahtani M., Aldohyan M.M., Alabdulsalam A.M., Alshammari M.S., Alajlan S.A., Binrokan A., Mohzari Y., Alrashed A. (2021). A Patent Review on the Therapeutic Application of Monoclonal Antibodies in COVID-19. Int. J. Mol. Sci..

[9] Alshrari A.S., Hudu S.A., Imran M., Asdaq S.M.B., Ali A.M., Rabbani S.I. (2022). Innovations and development of COVID-19 vaccines: A patent review. J. Infect. Public Health.

[10] Vishwakarma S., Panigrahi C., Barua S., Sahoo M., Mandliya S. (2022). Food nutrients as inherent sources of immunomodulation during COVID-19 pandemic. Lebensm. Wiss. Technol..

[11] Kumar A., Rai A., Khan M.S., Kumar A., Haque Z.U., Fazil M., Rabbani G. (2022). Role of herbal medicines in the management of patients with COVID-19: A systematic review and meta-analysis of randomized controlled trials. J. Tradit. Complement. Med..

[12] Derosa G., Maffioli P., D’Angelo A., Di Pierro F. (2021). A role for quercetin in coronavirus disease 2019 (COVID-19). Phytother. Res..

[13] Bernini R., Velotti F. (2021). Natural Polyphenols as Immunomodulators to Rescue Immune Response Homeostasis: Quercetin as a Research Model against Severe COVID-19. Molecules.

[14] Manjunath S.H., Thimmulappa R.K. (2021). Antiviral, immunomodulatory, and anticoagulant effects of quercetin and its derivatives: Potential role in prevention and management of COVID-19. J. Pharm. Anal..

[15] Diniz L.R.L., Souza M.T.S., Duarte A.B.S., Sousa D.P. (2020). Mechanistic Aspects and Therapeutic Potential of Quercetin against COVID-19-Associated Acute Kidney Injury. Molecules.

[16] DI Pierro F., Khan A., Bertuccioli A., Maffioli P., Derosa G., Khan S., Khan B.A., Nigar R., Ujjan I., Devrajani B.R. (2021). Quercetin Phytosome® as a potential candidate for managing COVID-19. Minerva Gastroenterol. Torino.

[17] Colunga Biancatelli R.M.L., Berrill M., Catravas J.D., Marik P.E. (2020). Quercetin and Vitamin C: An Experimental, Synergistic Therapy for the Prevention and Treatment of SARS-CoV-2 Related Disease (COVID-19). Front. Immunol..

[18] D’Andrea G. (2015). Quercetin: A flavonol with multifaceted therapeutic applications?. Fitoterapia.

[19] Dabbagh-Bazarbachi H., Clergeaud G., Quesada I.M., Ortiz M., O’Sullivan C.K., Fernández-Larrea J.B. (2014). Zinc ionophore activity of quercetin and epigallocatechin-gallate: From Hepa 1-6 cells to a liposome model. J. Agric. Food. Chem..

[20] Ansari M.A., Abdul H.M., Joshi G., Opii W.O., Butterfield D.A. (2009). Protective effect of quercetin in primary neurons against Abeta(1–42): Relevance to Alzheimer’s disease. J. Nutr. Biochem..

[21] Anand David A.V., Arulmoli R., Parasuraman S. (2016). Overviews of Biological Importance of Quercetin: A Bioactive Flavonoid. Pharmacogn. Rev..

[22] Yang D., Wang T., Long M., Li P. (2020). Quercetin: Its Main Pharmacological Activity and Potential Application in Clinical Medicine. Oxid. Med. Cell Longev..

[23] Grass Notices. https://www.cfsanappsexternal.fda.gov/scripts/fdcc/index.cfm?set=GRASNotices&id=341.

[24] Bhat I.U.H., Bhat R. (2021). Quercetin: A Bioactive Compound Imparting Cardiovascular and Neuroprotective Benefits: Scope for Exploring Fresh Produce, Their Wastes, and By-Products. Biology.

[25] Costela-Ruiz V.J., Illescas-Montes R., Puerta-Puerta J.M., Ruiz C., Melguizo-Rodríguez L. (2020). SARS-CoV-2 infection: The role of cytokines in COVID-19 disease. Cytokine Growth Factor Rev..

[26] Markoulaki D., Iordanou S., Koukios D., Christoldoulou I., Papadopoulos P., Timiliotou-Matsentidou C. (2022). Severe Multisystem Inflammatory Syndrome Associated with SARS-CoV-2 in a 31-Year-Old Male Patient: The First Clinical Case Report from the Republic of Cyprus. Cureus.

[27] Rondanelli M., Perna S., Gasparri C., Petrangolini G., Allegrini P., Cavioni A., Faliva M.A., Mansueto F., Patelli Z., Peroni G. (2022). Promising Effects of 3-Month Period of Quercetin Phytosome® Supplementation in the Prevention of Symptomatic COVID-19 Disease in Healthcare Workers: A Pilot Study. Life.

[28] Saeedi-Boroujeni A., Mahmoudian-Sani M.R. (2021). Anti-inflammatory potential of Quercetin in COVID-19 treatment. J. Inflamm. Lond..

[29] Aucoin M., Cooley K., Saunders P.R., Cardozo V., Remy D., Cramer H., Neyre Abad C., Hannan N. (2020). The effect of quercetin on the prevention or treatment of COVID-19 and other respiratory tract infections in humans: A rapid review. Adv. Integr. Med..

[30] Clinical Trial Database. https://www.clinicaltrials.gov/.

[31] Shohan M., Nashibi R., Mahmoudian-Sani M.R., Abolnezhadian F., Ghafourian M., Alavi S.M., Sharhani A., Khodadadi A. (2022). The therapeutic efficacy of quercetin in combination with antiviral drugs in hospitalized COVID-19 patients: A randomized controlled trial. Eur. J. Pharmacol..

[32] Margolin L., Luchins J., Margolin D., Margolin M., Lefkowitz S. (2021). 20-Week Study of Clinical Outcomes of Over-the-Counter COVID-19 Prophylaxis and Treatment. J. Evid. Based Integr Med..

[33] Di Pierro F., Iqtadar S., Khan A., Ullah Mumtaz S., Masud Chaudhry M., Bertuccioli A., Derosa G., Maffioli P., Togni S., Riva A. (2021). Potential Clinical Benefits of Quercetin in the Early Stage of COVID-19: Results of a Second, Pilot, Randomized, Controlled and Open-Label Clinical Trial. Int. J. Gen. Med..

[34] Di Pierro F., Derosa G., Maffioli P., Bertuccioli A., Togni S., Riva A., Allegrini P., Khan A., Khan S., Khan B.A. (2021). Possible Therapeutic Effects of Adjuvant Quercetin Supplementation Against Early-Stage COVID-19 Infection: A Prospective, Randomized, Controlled, and Open-Label Study. Int. J. Gen. Med..

[35] Önal H., Arslan B., Üçüncü Ergun N., Topuz Ş., Yilmaz Semerci S., Kurnaz M.E., Molu Y.M., Bozkurt M.A., Süner N., Kocataş A. (2021). Treatment of COVID-19 patients with quercetin: A prospective, single center, randomized, controlled trial. Turk. J. Biol..

[36] Xia L., Shi Y., Su J., Friedemann T., Tao Z., Lu Y., Ling Y., Lv Y., Zhao R., Geng Z. (2021). Shufeng Jiedu, a promising herbal therapy for moderate COVID-19: Antiviral and anti-inflammatory properties, pathways of bioactive compounds, and a clinical real-world pragmatic study. Phytomedicine.

[37] Feng Y., Zhu B., Liu Y., Liu Y., Zhou G., Yang L., Liu L., Ren J., Hou Y., Yu H. (2022). Yindan Jiedu granules exhibit anti-inflammatory effect in patients with novel Coronavirus disease (COVID-19) by suppressing the NF-κB signaling pathway. Phytomedicine.

[38] Imran M., Khan S.A., Abida’ Alshrari A.S., Eltahir Mudawi M.M., Alshammari M.K., Harshan A.A., Alshammari N.A. (2022). Small molecules as kinetoplastid specific proteasome inhibitors for Leishmaniasis: A patent review from 1998 to 2021. Expert Opin. Ther. Pat..

[39] Imran M., Khan S.A., Alshammari M.K., Alreshidi M.A., Alreshidi A.A., Alghonaim R.S., Alanazi F.A., Alshehri S., Ghoneim M.M., Shakeel F. (2021). Discovery, Development, Inventions, and Patent Trends on Mobocertinib Succinate: The First-in-Class Oral Treatment for NSCLC with EGFR Exon 20 Insertions. Biomedicines.

[40] Imran M., Khan S.A., Alshammari M.K., Alqahtani A.M., Alanazi T.A., Kamal M., Jawaid T., Ghoneim M.M., Alshehri S., Shakeel F. (2022). Discovery, Development, Inventions and Patent Review of Fexinidazole: The First All-Oral Therapy for Human African Trypanosomiasis. Pharmaceuticals.

[41] Imran M., Alsharari A.S., Thabet H.K., Abida, Bakht A.M. (2021). Synthetic molecules as DprE1 inhibitors: A patent review. Expert Opin. Ther. Pat..

[42] Imran M., Asdaq S.M.B., Khan S.A., Unnikrishnan Meenakshi D., Alamri A.S., Alsanie W.F., Alhomrani M., Mohzari Y., Alrashed A., AlMotairi M. (2021). Innovations and Patent Trends in the Development of USFDA Approved Protein Kinase Inhibitors in the Last Two Decades. Pharmaceuticals.

[43] Niedzwiecki A., Rath M.W., Ivanov V.O., Goc A. (2021). Pharmaceutical Micronutrient Composition, and Its Use to Simultaneously Inhibit Multiple Cellular Mechanisms of Infectivity Caused by Coronavirus, Its Variants and Mutants. U.S. Patent.

[44] Khalil I.T.I., Abd El-Latif S.A.M. (2021). A New and Safe Pharmaceutical Preparation for Prophylaxis and Treatment of Respiratory Viral Infections, Especially Corona Viruses. PCT Patent.

[45] Hofleitner P. (2021). Composition for Treating Viral Infections. PCT Patent.

[46] Hahn N. (2021). Nutraceutical Composition. PCT Patent.

[47] Salunke P.P., Salunke V.P., Patil P.E. (2021). A Composition for Management of COVID-19 and Associated Disorders. PCT Patent.

[48] Spadavecchia J., Giousuè Balzanelli M., Derrien M. (2021). Composition for the Prevention or Treatment of COVID-19. PCT Patent.

[49] Suzman P., Fisherman J., Lunsmann W. (2021). Methods and Compositions for Treating Viral Respiratory Infections. PCT Patent.

[50] Voelkel N.F., Magolske C. (2022). Method and Composition for Treating Coronavirus, Influenza, and Acute Respiratory Distress Syndrome. U.S. Patent.

[51] Schack D.M., Campbell A.W. (2021). Anthocyanin and Quercetin Based Formulations for Improved Respiratory Health. U.S. Patent.

[52] Margolin L. (2021). Compositions and Methods for Dietary Enhancement of Immune System Function. U.S. Patent.

[53] Meydani S.N., Mozaffarian D. (2021). Nutritional Supplement to Combat COVID-19 and Aid Vaccination. U.S. Patent.

[54] Hazan S. (2021). Methods of Preventing and Treating COVID-19 Infection. U.S. Patent.

[55] Li Y., Miao L., Zhao W., Xu Z., Pan X., Zhou W., Sun M., Zuo Y. (2021). Small- Molecule Inhibitor for Blocking Combination of COVID-19 Spinous Protein and Human Angiotensin Converting Enzyme 2 and Application Thereof. Chinese Patent.

[56] Su W., Yang Z., Zhong N., Ma Q., Li R., Li P., Peng W., Wu H., Shi R., Wang Y. (2021). Herba Hyperici Japonici Extract and Application Thereof in Preparation of Novel Coronavirus Resistant Drug. Chinese Patent.

[57] Feng Y., Xu C., Gao J., Ge Q., Lu Y., Wu Z., Zhang Y. (2020). Application of Quercetin to Preparation of Anti-Novel Coronavirus Drugs. Chinese Patent.

[58] Nezami M. (2021). Compositions and Methods for Preventing and/or Treating Viral Infection. PCT Patent.

[59] Newman R.A., Addington O.C. (2020). Method and Compositions for Treating Coronavirus Infection. U.S. Patent.

[60] Barnhill S. (2022). Antiviral Compositions and Methods for Their Use. PCT Patent.

[61] St. John A. (2022). Targeting Immune Pathologies Induced by Highly Pathogenic Coronaviruses. PCT Patent.

[62] Yimam M., Jiao P., Horm T., Brownell L., Hong M., O’Neal A., Jia Q. (2022). Aloe Based Compositions Comprising Polysaccharides and Polyphenols for Regulation of Homeostasis of Immunity. U.S. Patent.

[63] Kelesidis T., Arumugaswami V., Garcia (2022). Compositions and Methods for Inhibiting and Treating Coronavirus Infections. PCT Patent.

[64] Ghaderi L. (2022). Compositions and Methods for Inducing Biological Mimicry in a Mammal for the Prevention and/or Treatment of COVID-19 and Other Diseases. U.S. Patent.

[65] Robitaille M., Laurin P., Gagnon L., Cesari F. (2021). Pharmacotherapeutic Doses of Hesperidin or Related Bioflavonoids to Adress Infectious and/or Inflammatory Diseases. PCT Patent.

[66] Hickok S.S. (2021). Improved Immunomodulator Compositions and Viral Pathogen Treatments. PCT Patent.

[67] Obiso R., Newman R., Addington O. (2021). Extract Containing Oleandrin and Method of Production Thereof. PCT Patent.

[68] Dite G.S., Murphy N.M., Allman R. (2021). Methods of Assessing Risk of Developing a Severe Response to Coronavirus Infection. PCT Patent.

[69] Adnot S., Trottein F., Bernard D. (2021). Method to Treat a Pathogen Lung Infection. PCT Patent.

[70] Zhang H. (2021). Compositions and Methods for Preventing and/or Treating Microbial Infections. PCT Patent.

[71] Weiguang W., Vinodh K. (2021). Topical Disulfiram for Treating Viral Infections. PCT Patent.

[72] Ichim T., Lin F., Pingle S., Ashili S. (2021). Augmentation of Cell Therapy Efficacy by Inhibition of Complement Activation Pathways. U.S. Patent.

[73] O’heeron P., Ichim T. (2021). Reduction of Cytokine Storm and Pathological Inflammation Including Caused by Coronavirus Using Sphagnum and Extracts Thereof. PCT Patent.

[74] Fliri A. (2021). Drug Combinations for Inhibiting Infectivity of Influenza and Corona Viruses. PCT Patent.

[75] Lecanu L. (2021). Pannexin-1 Inhibitors for the Treatment of SARS-COV-2 Infected COVID-19 Patients with or without an Associated Acute Respiratory Syndrome. PCT Patent.

[76] Finzi E. (2021). Zinc for Treating COVID-19. PCT Patent.

[77] Hoag G.G., Salerno J. (2021). Method for Treating Viral and Bacterial Infection Through Inhalation Therapy. PCT Patent.

[78] Parkin J., Wells J.M., Dransfield M. (2021). Methods Involving Neutrophil Elastase Inhibitor Alvelestat for Treating Coronavirus Infection. PCT Patent.

[79] Vecht-Lifshitz S.E. (2021). Pharmaceutical Compositions for Treating Corona Virus Disease. PCT Patent.

[80] Mcmahon C.D., Watson D. (2021). Viral Treatments Involving Manuka Honey and Components Thereof. PCT Patent.

[81] Newman R.A., Addington O.C., Obiso R.J. (2021). Method and Compositions for Treating Coronavirus Infection. PCT Patent.

[82] Newman R.A., Addington O.C., Obiso R. (2021). Method and Compositions for Treating Coronavirus Infection. PCT Patent.

[83] Ichim T., O’heeron P. (2021). Fibroblast Mediated Expansion and Augmentation of Immune Regulatory Cells for Treatment of Acute Respiratory Distress Syndrome (ARDS). PCT Patent.

[84] Budak G.G., Budak M. (2021). The Natural Antiviral and Anti-Inflammatory Compound Consist of Bioflavonoids Which Extracted from Papaver Rhoeas Red Petals. PCT Patent.

[85] Davidoff A. (2021). Methods of Treating Viral Infections and Health Consequences. PCT Patent.

[86] Popov T., Josling P.D. (2021). Compositions and Applications Thereof. PCT Patent.

[87] Xiao Z., Xiao S., Peng G., He Z. (2021). Formulations Comprising Botanical Extracts. Australian Patent.

[88] Jain S. (2021). Method and System for Detecting and Treating Exposure to an Infectious Pathogen. PCT Patent.

[89] Winchester H. (2021). Methods of Diagnosing Risk of Serious Symptoms from COVID-19 Infection. U.S. Patent.

[90] Kang S., Kwon J., Jung Y., Choi S., Lee Y. (2021). SARS-CoV-2 Composition for the Prevention or Treatment of SARS-CoV-2 Infection Comprising the Extract of Agrimonia pilosa as an Active Ingredient. Korean Patent.

[91] Chung J.Y., Lee P.G., Kim Y.P., Lee J.H., Lee J.J. (2021). Coronavirus Therapeutic Agent Comprising Zanthoxylum piperitum Leaf Extract as Active Ingredient. PCT Patent.

[92] Ye D., Lu Y., Zhou J., Lin Y., Li H., Deng C. (2020). Medicinal and Edible Traditional Chinese Medicine Preparation and Application Thereof. Chinese Patent.

[93] Rincome U., Rincome S. (2021). Fermented—Concentrated Extraction Method of Houttuynia cordata for Medical Using. PCT Patent.

[94] Niedzwiecki A., Rath M.W., Ivanov V., Goc A. (2022). Micronutrient Combination to Inhibit Coronavirus Cell Infection. U.S. Patent.

[95] Ichim T., Lin F., Pingle S., Ashili S. (2021). Treatment of Acute Respiratory Distress Syndrome by T Regulatory Cells. U.S. Patent.

[96] Stafford V.R. (2021). Method to Mitigate Morbidity and Mortality in Virally Induced forms of ACE2 Receptor Pathology Progressing to SARS or ARDS. U.S. Patent.

